# Genome-Scale Assessment of Age-Related DNA Methylation Changes in Mouse Spermatozoa

**DOI:** 10.1371/journal.pone.0167127

**Published:** 2016-11-23

**Authors:** Norio Kobayashi, Hiroaki Okae, Hitoshi Hiura, Hatsune Chiba, Yoshiki Shirakata, Kenshiro Hara, Kentaro Tanemura, Takahiro Arima

**Affiliations:** 1 Department of Informative Genetics, Tohoku University Graduate School of Medicine, 2–1 Seiryo-cho, Aoba-ku, Sendai, 980–8575, Japan; 2 Laboratory of Animal Reproduction and Development, Graduate School of Agricultural Science, Tohoku University, 1–1 Amamiya-machi, Tsutsumidori, Aoba-ku, Sendai, 981–8555, Japan; Massachusetts General Hospital, UNITED STATES

## Abstract

DNA methylation plays important roles in the production and functioning of spermatozoa. Recent studies have suggested that DNA methylation patterns in spermatozoa can change with age, but the regions susceptible to age-related methylation changes remain to be fully elucidated. In this study, we conducted genome-scale DNA methylation profiling of spermatozoa obtained from C57BL/6N mice at 8 weeks (8w), 18 weeks (18w) and 17 months of age (17m). There was no substantial difference in the global DNA methylation patterns between 18w and 17m samples except for a slight increase of methylation levels in long interspersed nuclear elements in the 17m samples. We found that maternally methylated imprinting control regions (mICRs) and spermatogenesis-related gene promoters had 5–10% higher methylation levels in 8w samples than in 18w or 17m samples. Analysis of individual sequence reads suggested that these regions were fully methylated (80–100%) in a subset of 8w spermatozoa. These regions are also known to be highly methylated in a subset of postnatal spermatogonia, which might be the source of the increased DNA methylation in 8w spermatozoa. Another possible source was contamination by somatic cells. Although we carefully purified the spermatozoa, it was difficult to completely exclude the possibility of somatic cell contamination. Further studies are needed to clarify the source of the small increase in DNA methylation in the 8w samples. Overall, our findings suggest that DNA methylation patterns in mouse spermatozoa are relatively stable throughout reproductive life.

## Introduction

Spermatozoa develop from male primordial germ cells (PGCs). PGCs arise from proximal epiblast cells and migrate to the genital ridges, where they differentiate into mitotically arrested prospermatogonia (PSG) [1]. After birth, PSG resume proliferation and give rise to spermatogonial stem cells (SSCs), which ensure continual production of spermatozoa throughout life [2]. In male mice, mature spermatozoa appear at about 5 weeks after birth. The rate of spermatogenesis reaches adult levels by 11 weeks and starts to decline from 16 months onward [3].

DNA methylation is dynamically regulated in PGCs and PSG. Migrating PGCs undergo global DNA demethylation that includes erasure of genome imprints [4, 5]. Thereafter, *de novo* methylation occurs and ~80% of CpG cytosines are methylated in PSG [6, 7]. Global *de novo* methylation is essential for meiotic progression, retrotransposon silencing and the establishment of paternally methylated imprinting control regions (pICRs) [6–8]. After birth, some regions further exhibit substantial methylation changes in postnatal spermatogonia [7]. A recent study revealed that maternally methylated imprinting control regions (mICRs) and spermatogenesis-related gene promoters are transiently methylated in a subset of postnatal spermatogonia, whereas these regions are hypomethylated in adult SSCs [9].

DNA methylation patterns are stably maintained during differentiation of adult SSCs into spermatozoa. In spite of the stability, many studies have suggested that various environmental and physiological factors, including nutrition [10], exercise [11], psychological stress [12, 13] and advanced age [14–16], can alter DNA methylation patterns in spermatozoa. Some of these studies have proposed that DNA methylation changes in spermatozoa can be inherited by subsequent generations. In particular, epidemiological studies have revealed that advanced paternal age increases the risk of psychiatric disorders such as autism, schizophrenia and bipolar disorders in offspring [17]. Recent human and mouse studies have also suggested that advanced age-induced DNA methylation changes in spermatozoa might cause psychiatric disorders in offspring [15, 16]. However, the reproducibility and robustness of these findings have hardly been assessed in independent studies. Thus, further studies are needed to verify the causal relationships among advanced paternal age, DNA methylation in spermatozoa and psychiatric disorders in offspring.

To obtain further insights into age-related DNA methylation changes in spermatozoa, we conducted a genome-scale DNA methylation analysis of spermatozoa collected from early postpubertal (8-week-old: 8w) and aged (17-month-old: 17m) mice, using 18-week-old (18w) mice as a reference.

## Material and Methods

### Mouse spermatozoa preparation

All animal studies were conducted following the guidelines of Tohoku University Institutional Animal Care and Use Committee approved this study (2016–049). C57BL/6N mice were purchased from Japan SLC, Inc. and housed at 24°C on a 12:12 h light-dark cycle. Spermatozoa were collected from the cauda epididymides of mice euthanized at 8 weeks, 18 weeks and 17 months of age. The spermatozoa were purified using the swim-up method. Briefly, the cauda epididymidis was cut using microspring scissors and squeezed. The spermatozoa suspension was transferred into a volume of 500 μl of human tubal fluid (HTF) medium (Shigma-Aldrich Japan, Tokyo, Japan) [18]. After incubation for 60 min at 37°C under 5% CO_2_ in humidified air, spermatozoa swimming toward the upper layer of the medium were collected (400 μl) and thereafter washed two times with PBS. We confirmed the absence of somatic cells in the isolated spermatozoa using an LSM-710 confocal laser microscope (Carl Zeiss, Jena, Germany).

### Reduced representation bisulfite sequencing (RRBS)

The spermatozoa were suspended in a lysis buffer (0.14 mM β-mercaptoethanol, 0.24 mg/ml proteinase K, 150 mM NaCl, 10 mM Tris-HCl [pH 8.0], 10 mM EDTA [pH 8.0] and 0.1% SDS) and incubated at 55°C overnight. Then sperm genomic DNA was isolated using phenol/chloroform extraction and ethanol precipitation as described previously [19]. RRBS libraries were also generated as previously reported [20]. Briefly, 20 ng genomic DNA was subjected to MspI digestion (NEB, Beverly, MA, USA). end repair/dA-tailing reaction using Klenow 3’-5’ exo- (NEB) and ligation by Illumina sequencing adapters using T4 DNA ligase (NEB). Then 150–350 bp fragments were gel-excised using NuSieve 3:1 agarose (Lonza Japan, Tokyo, Japan) and a MinElute Gel Extraction kit (QIAGEN, Valencia, CA, USA). The fragments were treated with sodium bisulphite using an EZ DNA Methylation-Gold Kit (Zymo Research, Orange, CA, USA). Library amplification and indexing were performed with KAPA HiFi HotStart Uracil+ ReadyMix (2×) (Kapa Biosystems, Woburn, MA). The PCR amplification was carried out as follows: an initial denaturation at 95°C for 2 min, 13 cycles of 98°C for 20 sec, 65°C for 30 sec and 72°C for 30 sec and a final 1 min extension at 72°C. The RRBS libraries were purified using Agencourt AMPure XP (Beckman Coulter, Brea, CA, USA) and thereafter quantified with a Kapa Library Quantification Kit (Kapa Biosystems), and were sequenced on an HiSeq 2500 platform (Illumina, CA, USA) with 100-bp single-end reads using a TruSeq SR Cluster Kit v3-cBot-HS and TruSeq SBS Kit v3-HS (Illumina). Sequenced reads were processed using an Illumina standard base-calling pipeline (v1.8.2) and the index and adapter sequences were removed. The first and last 4 bases were trimmed and the resulting reads were aligned to Mouse Genome Build 37 (mm9) using Bismark (v.0.9.0) [21] with default parameters. The methylation level of each cytosine was calculated using the Bismark methylation extractor. We analyzed only CpG cytosines covered with ≥5 reads.

### Bio-combined bisulfite restriction analysis (Bio-COBRA)

We confirmed *Plagl1* mICR, *Grb10* mICR, and *H19* pICR methylation levels using Bio-COBRA. Genomic DNA was treated with sodium bisulfite using an EZ DNA Methylation-Gold Kit (Zymo Research) and amplified by PCR (TaKaRa EpiTaq HS; Takara Biomedical, Otsu, Japan), according to the manufacturer’s protocol. The PCR reaction was performed in a volume of 20 μl with the following conditions: 98°C for 10 sec, followed by 40 cycles of denaturation at 98°C for 10 sec, annealing to the *Plagl1*, *Grb10* or *H19* primer at 57°C, 52°C or 52°C, respectively, for 30 sec, and extension at 72°C for 30 sec, followed by a final 5 min extension at 72°C. Of the 20 μl PCR products was digested with 5 units of BstUI (NEB) for *Plagl1* or *Grb10* and AciI (NEB) for *H19* at 60°C or 37°C, respectively, for 4 h, according to conditions specified by the manufacturer. The digested PCR products were visualized on D1000 ScreenTape using the Agilent 2200 TapeStation instrument (Agilent Technologies, Santa Clara, CA, USA). Undigested and digested fragments corresponded to unmethylated and methylated DNA, respectively. We used the following oligonucleotide primers: 5’-GGTATTTAGGAGATTTTGGTTTTG-3’ (forward primer) and 5’- ATCCCAACCCAAACTAAATAACAA-3’ (reverse primer) for *Plagl1*, 5’-GTTTATTATTTGGATTATTGTAGA-3’ (forward primer) and 5’- CCRAATTCRAAAACTATCCACTAAC-3’ (reverse primer) for *Grb10*, and 5’-GGGGAGAAAATTTAATTAGTTGTAAT-3’ (forward primer) and 5’- CACATACATTTTCTAAACTAATACCTC-3’ (reverse primer) for *H19*.

### Annotations of genomic regions

Annotations of Refseq genes and repeat sequences were downloaded from the UCSC Genome Browser. Refseq genes encoding microRNAs or small nucleolar RNAs were excluded from our analyses. Promoters were defined as regions 500 bp upstream and downstream from transcription start sites (TSSs) of Refseq transcripts. Gene bodies were defined as transcribed regions of Refseq transcripts except for promoters. For calculation of the mean methylation levels, we analyzed promoters containing ≥5 CpG cytosines with sufficient coverage for calculation of the methylation levels. Regions and names of the 15 ICRs were defined as previously reported [4]. Similarly, we considered only ICRs containing ≥5 CpG cytosines. Gene ontology (GO) analyses were performed using the Database for Annotation, Visualization and Integrated Discovery (DAVID) [22].

### Graphical presentation

Methylation levels of CpG cytosines were visualized using Integrative Genomics Viewer (IGV) software (http://www.broadinstitute.org/igv/). Line and bar charts were generated using the ggplot2 package in R (http://www.R-project.org/). Hierarchical clustering was performed using Cluster 3.0 software (http://bonsai.hgc.jp/~mdehoon/software/cluster/software.htm) and the results were visualized with the TreeView program (https://sourceforge.net/projects/jtreeview/) [23].

### Statistical analysis of the data

All statistical analyses were performed using R. The differentially methylated promoters were defined as follows: absolute methylation changes ≥5% and *P* < 0.05 (Welch-ANOVA [24] with Benjamini-Hochberg (BH) correction [25]). The Games-Howell test was used to identify sample pairs with significant methylation differences (*P* < 0.05).

### Accession number

Sequence data have been deposited in DDBJ/GenBank/EMBL under the accession number DRA004602.

## Results

### Stability of global DNA methylation in spermatozoa

To determine age-related DNA methylation changes in spermatozoa, we performed RRBS of spermatozoa collected from mice aged 8w (n = 7), 18w (n = 3) and 17m (n = 7) (S1 Table). A total of 1,183,572 CpG cytosines were covered in all the samples and used for the following analysis. We first compared the DNA methylation patterns of promoters, gene bodies and their neighboring regions (S1 Fig). In all samples, the typical hypomethylated pattern of promoters was observed. We also compared mean methylation levels of CpG cytosines in various genomic features, including promoters, exons, introns, intergenic regions and retrotransposons (Table 1). The mean methylation levels were nearly identical among all samples, though 8w samples had slightly but significantly lower methylation levels (~3%) of introns, intergenic regions and retrotransposons compared to 18w and 17m samples. We also found that the mean methylation levels of long interspersed nuclear elements (LINEs) gradually increased with age (80.0% at 8w, 82.0% at 18w and 83.1% at 17m). The methylation changes in LINEs were very small but statistically significant (*P* < 0.001, Games-Howell test). We further analyzed LINE-1 (L1) elements with sufficient coverage (≥500 CpG cytosines). The mean methylation levels of most of the L1 elements slightly but significantly increased with age (Table 1). Although some genomic features had slight methylation differences, the global DNA methylation levels were very similar among 8w, 18w and 17m samples.

**Table 1:** Stability of DNA methylation levels of various genomic features. Mean methylation levels (%) of CpG cytosines in promoter, exon, intron, intergenic regions LINE, LTR, SINE and L1 elements. The evolutionary ages of the L1 elements [35] are indicated. Data are shown as mean ± standard error (SE). Different letters indicate statistically significantly methylation differences (P < 0.05).

Genomic Regions				8w				18w				17m			
	Promoter			1.08	±	0.02	^a^	1.08	±	0.02	^a^	1.00	±	0.02	^a^
	Exon			14.26	±	0.08	^a^	14.37	±	0.02	^a^	14.33	±	0.03	^a^
	Intron			21.93	±	0.09	^b^	22.93	±	0.02	^a^	22.84	±	0.03	^a^
	Intergenic			37.39	±	0.17	^b^	38.46	±	0.08	^a^	38.51	±	0.06	^a^
	LINE			80.04	±	0.19	^c^	82.00	±	0.10	^a^	83.06	±	0.14	^b^
	LTR			67.16	±	0.30	^b^	68.41	±	0.13	^a^	68.77	±	0.12	^a^
	SINE			69.26	±	0.19	^b^	71.70	±	0.06	^a^	71.43	±	0.04	^a^
Evolutionary ages		L1 elements		8w				18w				17m			
	<1.5 million years		L1MdT	87.19	±	0.17	^c^	88.84	±	0.09	^a^	90.21	±	0.11	^b^
			L1MdA	83.08	±	0.21	^c^	85.20	±	0.18	^a^	86.41	±	0.17	^b^
			L1MdGf	85.67	±	0.35	^c^	87.02	±	0.14	^a^	88.62	±	0.17	^b^
	1.5–6 million years		L1MdF3	79.82	±	0.29	^c^	81.67	±	0.09	^a^	82.51	±	0.15	^b^
			L1MdF2	81.64	±	0.18	^c^	83.48	±	0.10	^a^	84.72	±	0.20	^b^
			L1MdF	80.15	±	0.20	^c^	81.52	±	0.02	^a^	83.68	±	0.25	^b^
			L1VL1	75.95	±	0.36	^c^	77.36	±	0.16	^a^	78.39	±	0.14	^b^
	>6 million years		L1_Mus1	79.86	±	0.15	^b^	82.02	±	0.30	^a^	82.66	±	0.15	^a^

### Identification of differentially methylated promoters

**Fig 1:**
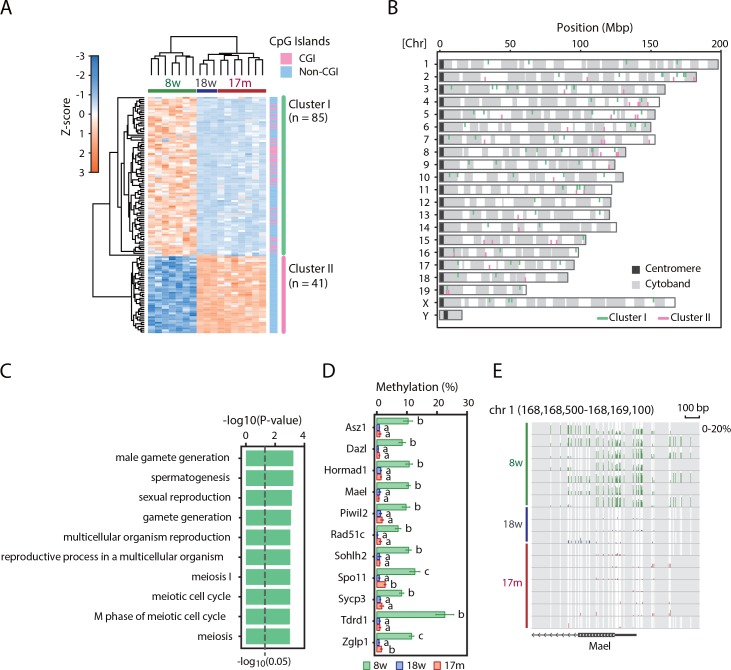
Identification of differentially methylated promoters. (A) Hierarchical clustering of differentially methylated promoters. Z-scored methylation levels are color-coded as shown. CGI and non-CGI promoters are also indicated. (B) Chromosome distribution of the differentially methylated promoters. (C) GO analysis of the promoters in Cluster I. Statistically significant (*P* < 0.05) GO terms are indicated with BH-corrected *P*-values. (D) Methylation levels of the promoters in Cluster I. Data are shown as mean ± SE. Different letters indicate statistically significantly methylation differences (*P* < 0.05). (E) Methylation pattern of the *Mael* promoter. The vertical axis indicates the methylation levels (%).

RRBS targets CpG-rich regions such as promoters and CpG islands, and promoter methylation is crucial for the regulation of gene expression. Therefore, we focused on the methylation levels of the promoters. We identified 126 promoters with absolute methylation changes ≥5% and statistically significant differences (*P* < 0.05, Welch-ANOVA with BH correction), and they were widely distributed across the genome (Fig 1A and 1B). These differentially methylated promoters were clearly divided into two clusters according to their methylation patterns (Fig 1A). The promoters in Cluster I had higher methylation levels in 8w samples than in 18w and 17m samples, and about half of them overlapped CpG islands (CGIs). On the other hand, the promoters in Cluster II had an opposite methylation pattern, and none of them overlapped CGIs. GO analysis revealed that promoters in Cluster I were significantly enriched in spermatogenesis-related GO terms such as male gamete generation and the meiotic cell cycle (Fig 1C). The spermatogenesis-related gene promoters had methylation levels that were 5–10% higher in 8w samples than in 18w and 17m samples (Fig 1D and 1E). The promoters in Cluster II were significantly enriched in cell projection-related gene promoters, while only 5 genes were annotated with this GO term (S2A Fig). These promoters showed 5–20% lower methylation levels in 8w samples than in 18w and 17m samples (S2B Fig).

**Fig 2:**
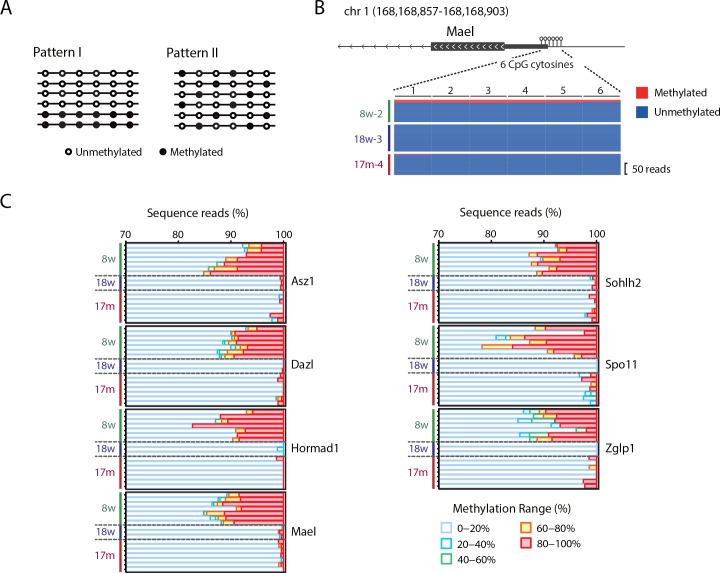
Heterogeneity of methylation levels of spermatogenesis-related promoters in 8w spermatozoa. (A) Heterogeneity of DNA methylation levels in spermatozoa. Methylation patterns of individual sequence reads are useful to verify whether subsets of spermatozoa have full methylation levels (pattern I) or not (pattern II). Note that the mean methylation levels are the same for both patterns. (B) Heterogeneity of the *Mael* promoter. *Mael* methylation patterns of 8w, 18w and 17m samples are shown. The region contains 6 CpG cytosines. The number of reads is indicated on the right side. Each line represents one read. Blue, unmethylated CpG cytosine; red, methylated CpG cytosine. (C) Analysis of individual sequence reads mapped to spermatogenesis-related gene promoters. Sequence reads were classified into five groups according to their methylation levels. The distribution of the methylation levels is shown as stacked bar charts.

We next focused on the methylation patterns of individual sequence reads, which are useful to determine whether a subset of spermatozoa have full methylation levels (pattern I in Fig 2A) or not (pattern II in Fig 2A). We used only reads containing at least 5 CpG cytosines and analyzed differentially methylated promoters where ≥20 reads were mapped. Seven spermatogenesis-related gene promoters were successfully analyzed. We found that 5–15% of reads in these promoters were >80% methylated and most of the other reads were <20% methylated (Fig 2B and 2C), supporting pattern I in Fig 2A. Although we carefully purified the spermatozoa, it was difficult to completely exclude the possibility of somatic cell contamination. As spermatogenesis-related promoters are frequently hypermethylated in somatic cells, the higher methylation levels of these regions in 8w samples could be attributable to somatic cell contamination. If so, the highly methylated reads must be excluded from the analysis. Considering that ≥60% methylated reads were specifically observed in 8w samples (Fig 2C), we recalculated the methylation levels of the spermatogenesis-related promoters without these ≥60% methylated reads. After the removal of the ≥60% methylated reads, most of these regions had similar methylation levels in all samples (S5A Fig). This result suggested that if our spermatozoa samples were contaminated with somatic cells, spermatogenesis-related promoters might not be differentially methylated among 8w, 18w and 17m spermatozoa.

### Differential methylation of mICRs

**Fig 3:**
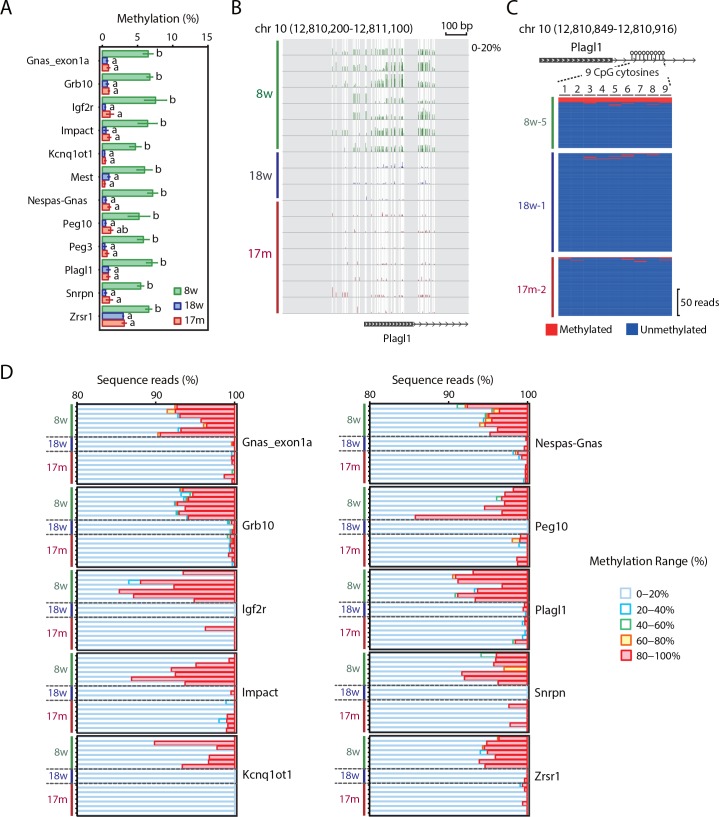
Heterogeneity of methylation levels of mICRs in 8w spermatozoa. (A) Methylation levels of mICRs. Data are shown as mean ± SE. Different letters indicate statistically significant methylation differences (*P* < 0.05). (B) Methylation pattern of the *Plagl1* ICR. The vertical axis indicates the methylation levels (%). (C) Heterogeneity of *Plagl1* ICR. *Plagl1* ICR methylation patterns of 8w, 18w and 17m samples are shown. The region contains 9 CpG cytosines. The number of reads is indicated on the right. Blue, unmethylated CpG cytosine; red, methylated CpG cytosine. (D) Analysis of individual sequence reads. The vertical axis indicates the samples. Sequence reads were classified into five groups according to their methylation levels. The distribution of the methylation levels is shown as stacked bar charts.

The differentially methylated promoters (Fig 1A) included two mICRs, the *Gnas* and *Plagl1* ICRs, and therefore we analyzed the DNA methylation levels in other mICRs. Methylation levels were available for ten additional mICRs, and most of were 5–8% higher in 8w samples than in 18w and 17m samples (Fig 3A, 3B and S3 Fig). We analyzed individual reads as described above, and confirmed that 5–10% of reads in these promoters were >80% methylated whereas most of the other reads were <20% methylated (Fig 3C and 3D). The methylation levels of pICRs were not available in our RRBS data due to the lack of informative CpG cytosines. Thus, we analyzed the DNA methylation levels of the *H19* pICR using Bio-COBRA. The *H19* methylation levels were about 10% lower in 8w samples than in 18w and 17m samples (S4A and S4B Fig).

As with the spermatogenesis-related promoters, mICRs had similar methylation levels in all samples when the ≥60% methylated reads were excluded from the analysis (S5B Fig).

## Discussion

We found that mICRs and spermatogenesis-related gene promoters had 5–10% higher methylation levels in spermatozoa samples from 8w mice than in those from 18w and 17m mice. A recent study revealed that mICRs and spermatogenesis-related gene promoters were transiently methylated in a subset of postnatal spermatogonia, whereas these regions were hypomethylated in adult SSCs [9]. The methylation changes in 8w spermatozoa were very similar to those observed in postnatal differentiating (KIT^+^) spermatogonia [9]. Therefore, some of the postnatal spermatogonia with high methylation in mICRs and spermatogenesis-related gene promoters might contribute to early postpubertal spermatozoa. In contrast, mICRs and spermatogenesis-related gene promoters had very low methylation levels (~3% or less) in 18w and 17m spermatozoa samples, consistent with the unmethylated patterns of these regions in adult SSCs [9]. Our findings suggested that early postnatal spermatozoa with high methylation in mICRs and spermatogenesis-related gene promoters might not contribute to adult SSCs, ensuring the normal DNA methylation patterns in spermatozoa after the early postpubertal stage.

These data raised the interesting possibility that early postpubertal spermatozoa might be associated with a higher rate of birth defects or fertilization failure because genomic imprinting is essential for normal mouse development and spermatogenesis-related genes are required for normal spermatogenesis. A previous study reported that morphologically abnormal spermatozoa collected from young (7 weeks of age) BALB/c AnN mice produced a slightly lower number of 2-cell embryos after ICSI than those from older (6–10 months of age) mice [26]. It is uncertain whether the low developmental rate of ICSI embryos is applicable to C57BL/6N (the strain used in our study) because the proportion of morphologically abnormal spermatozoa is very low in C57BL/6N [27] compared to BALB/c AnN [26]. Further studies will be required to verify the relationships among the DNA methylation patterns, morphology and functioning of early postpubertal spermatozoa.

Intriguingly, gain of methylation at mICRs (e.g., *KCNQ1OT1*, *MEST*, *PLAGL1 and SNRPN*) and loss of methylation at pICRs (e.g., *H19*) are frequently observed in spermatozoa from infertile human patients [28, 29]. In addition, gain of methylation in spermatogenesis-related gene promoters, including *DAZL*, *PIWIl2* and *TDRD1*, is observed in spermatozoa and testicular samples from infertile human patients [30, 31]. We found that these infertility-related aberrant DNA methylation patterns were also observed spermatozoa from 8w mice. Although it is unclear why spermatozoa of infertile human patients and early postpubertal mice have similar DNA methylation alterations, there might be a common underlying mechanism. Therefore, we inferred that the first wave of spermatogenesis, which contributes to early postpubertal spermatozoa, could be used as a model for understanding the mechanism of infertility-related aberrant DNA methylation in human spermatozoa.

Milekic et al. recently reported that a significant loss of methylation in spermatozoa was observed in aged (12–14 months old) mice compared to young (3 months of age) mice [16]. In contrast, we found that global DNA methylation levels in spermatozoa were nearly identical in 18w and 17m mice except for the slight change in LINE methylation. This discrepancy might reflect the differences in the mouse strains used (C57BL/6N in our study, 129SvEv/Tac in the study by Milekic et al.), DNA methylation analysis techniques (RRBS in our study, methylation mapping analysis by paired-end sequencing (Methyl-MAPS) in the study by Milekic et al.), spermatozoa preparation methods (the spermatozoa preparation method is not provided in the study by Milekic et al) and/or age. Methyl-MAPS, which is based on restriction enzymes, covers more CpG sites than RRBS, but has lower resolution and is less quantitative. For better understanding of advanced age-related DNA methylation changes, it will be beneficial to compare various mouse strains at different ages using whole genome bisulfite sequencing, which is the current gold standard for DNA methylation analysis.

A recent study on human spermatozoa showed that the DNA methylation levels of LINEs were slightly higher in men aged >45 years than in those aged ≤45 years [15]. Consistently, we found that spermatozoa of 17m mice had slightly but significantly higher methylation levels of LINEs than those of 18w mice. It is unlikely that the slight increase in LINE methylation has deleterious effects on spermatozoa because DNA methylation-mediated silencing of LINEs is an important mechanism to maintain genome integrity [32, 33]. Though the functional implications of the age-related changes in LINE methylation are unclear, LINE methylation levels might be useful as a marker for aging of human and mouse spermatozoa.

A technical limitation of this study is that spermatozoa were analyzed in bulk. We could not determine whether multiple ICRs and spermatogenesis-related gene promoters were highly methylated in individual 8w spermatozoa. In addition, although we carefully purified the spermatozoa using the swim-up method and microscopically confirmed the absence of somatic cells, we could not completely exclude the possibility of somatic cell contamination. For example, cell debris or genomic DNA released from dead somatic cells might have been included in our spermatozoa samples. To address these problems, single-cell DNA methylation analysis will be useful [34].

In conclusion, DNA methylation patterns in mouse spermatozoa are relatively stable throughout reproductive life, which may underlie the continual production of fully competent spermatozoa throughout the reproductive life.

## Supporting Information

S1 Table Summary of RRBS. RRBS libraries of spermaotozoa were prepared from mice aged 8w (n = 7), 18w (n = 3) and 17m (n = 7). The CpG cytosines covered with ≥5 reads are indicated. Bisulfite conversion rates were >99% for all samples.
(PDF)

S1 Fig Stability of global DNA methylation levels. DNA methylation patterns of transcription start sites (TSS), gene bodies and their neighboring regions. Mean methylation levels of 8w (n = 7), 18w (n = 3) and 17m (n = 7) samples are shown. TES: transcription end sites.
(PDF)

S2 Fig Differentially methylated promoters in 18w and 17m spermatozoa. (A) GO analysis of the promoters in Cluster II. (B) Methylation levels of the promoters in Cluster II. Data are shown as mean ± SE. Different letters indicate statistically significant methylation differences (*P* < 0.05).
(PDF)

S3 Fig Methylation patterns of mICRs. The vertical axis indicates the methylation levels (%).
(PDF)

S4 Fig DNA methylation of *Plagl1* mICR, *Grb10* mICR and *H19* pICR by Bio-COBRA. (A) DNA methylation of *Plagl1* mICR, *Grb10* mICR and *H19* pICR in each age sample. *Plagl1*, *Grb10* and *H19* amplified by PCR were digested with BstUI or AciI. Kidney DNA was used as a control. (B) Methylation levels of *Plagl1* mICR, *Grb10* mICR and *H19* pICR. Data are shown as mean ± SE. Different letters indicate statistically significant methylation differences (*P* < 0.05).
(PDF)

S5 Fig Methylation levels of spermatogenesis-related promoters and mICRs without highly methylated reads. (A and B) Methylation levels of the spermatogenesis-related promoters and mICRs were calculated without ≥60% methylated reads. Data are shown as mean ± SE. Different letters indicate statistically significant methylation differences (*P* < 0.05).
(PDF)
