# Supplementary material for: Genome-Scale Assessment of Age-Related DNA Methylation Changes in Mouse Spermatozoa
Source: PLoS One. 2016 Nov 23;11(11):e0167127. doi: 10.1371/journal.pone.0167127 (PMC5120852; doi:10.1371/journal.pone.0167127)
Supplement: S1 Table — RRBS libraries of spermaotozoa were prepared from mice aged 8w (n = 7), 18w (n = 3) and 17m (n = 7). The CpG cytosines covered with ≥5 reads are indicated. Bisulfite conversion rates were >99% for all samples. (PDF) [file pone.0167127.s001.pdf]

Supplementary Table 1. Summary of RRBS.

Age	Sample	Total reads	Alinged reads	Mapping rate (%)	Conversion rate (%)	Number of CpG cytosines covered with ≥5 reads	Number of merged CpG cytosines covered with ≥5 reads
8w	8w-1	29,269,953	20,092,503	68.6	99.5	1,387,664	
	8w-2	32,720,765	22,261,574	68.0	99.5	1,374,085	
	8w-3	28,666,944	19,865,785	69.3	99.4	1,422,153	
	8w-4	17,743,426	11,590,885	65.3	99.3	1,370,159	
	8w-5	16,249,591	10,539,139	64.9	99.3	1,333,015	
	8w-6	18,047,909	11,757,073	65.1	99.3	1,393,367	
	8w-7	19,486,339	12,694,390	65.1	99.3	1,388,393	
18w	18w-1	32,670,626	21,631,349	66.2	99.4	1,404,686	
	18w-2	34,196,751	22,476,161	65.7	99.4	1,395,049	1,183,572
	18w-3	28,569,568	18,675,326	65.4	99.4	1,419,527	
17m	17m-1	21,874,619	14,673,352	67.1	99.5	1,389,961	
	17m-2	20,246,339	13,516,012	66.8	99.4	1,328,112	
	17m-3	24,014,559	16,265,973	67.7	99.6	1,363,163	
	17m-4	18,762,100	12,181,794	64.9	99.3	1,406,687	
	17m-5	19,607,144	12,650,786	64.5	99.3	1,402,991	
	17m-6	18,871,569	12,112,696	64.2	99.4	1,376,972	
	17m-7	18,928,468	12,246,994	64.7	99.3	1,358,881	

RRBS libraries of spermaotozoa were prepared from mice aged 8w (n = 7), 18w (n = 3) and 17m (n = 7). The CpG cytosines covered with ≥5 reads are indicated. Bisulfite conversion rates were >99% for all samples.
