# Supplementary figures and images for: Genome-Scale Assessment of Age-Related DNA Methylation Changes in Mouse Spermatozoa
Source: PLoS One. 2016 Nov 23;11(11):e0167127. doi: 10.1371/journal.pone.0167127 (PMC5120852; doi:10.1371/journal.pone.0167127)

# Supplementary Figure 1

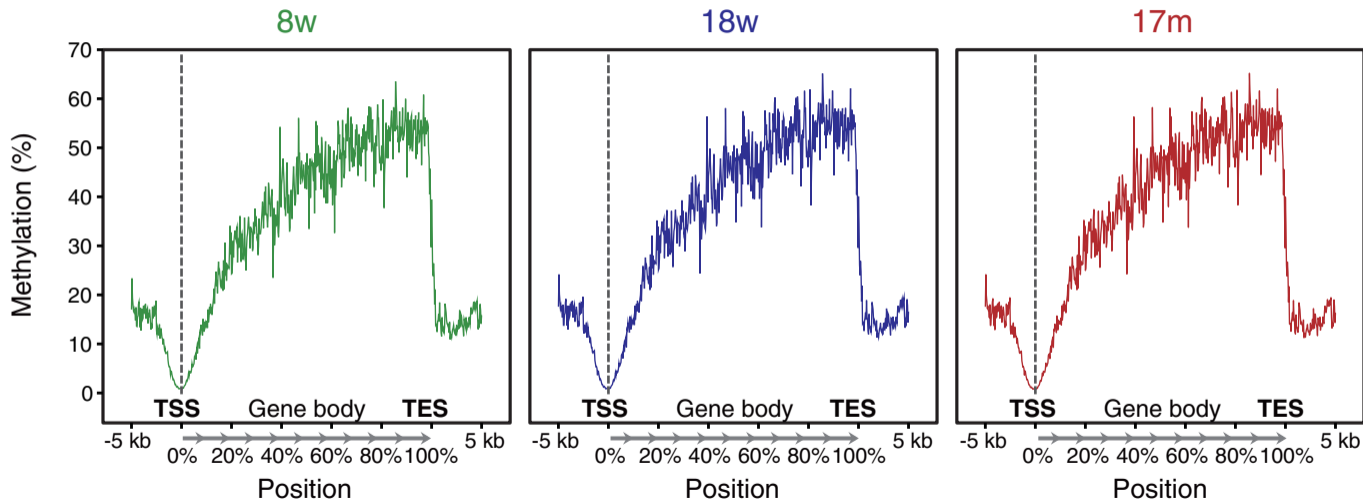

Supplement: S1 Fig — DNA methylation patterns of transcription start sites (TSS), gene bodies and their neighboring regions. Mean methylation levels of 8w (n = 7), 18w (n = 3) and 17m (n = 7) samples are shown. TES: transcription end sites. (PDF) [file pone.0167127.s002.pdf]

# Supplementary Figure 2

A

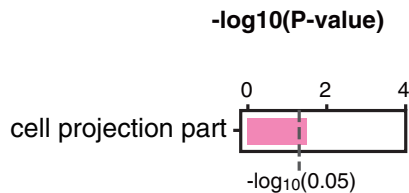

B

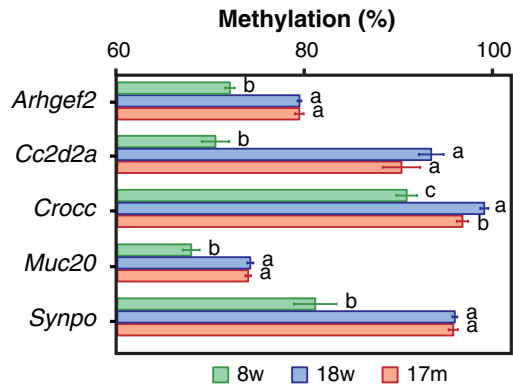

Supplement: S2 Fig — (A) GO analysis of the promoters in Cluster II. (B) Methylation levels of the promoters in Cluster II. Data are shown as mean ± SE. Different letters indicate statistically significant methylation differences (P < 0.05). (PDF) [file pone.0167127.s003.pdf]

# Supplementary Figure 3\_1

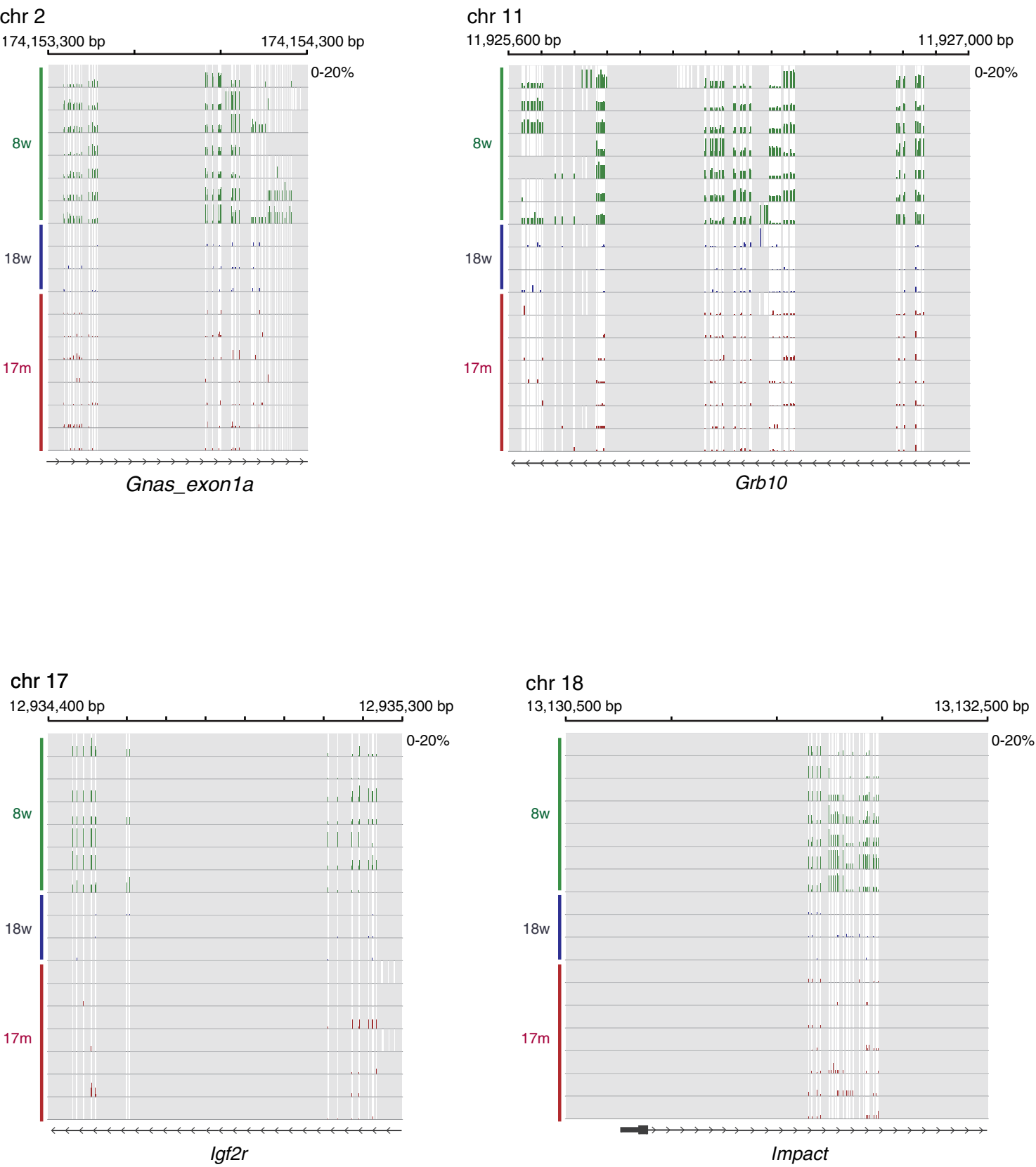

# Supplementary Figure 3\_2 (continued)

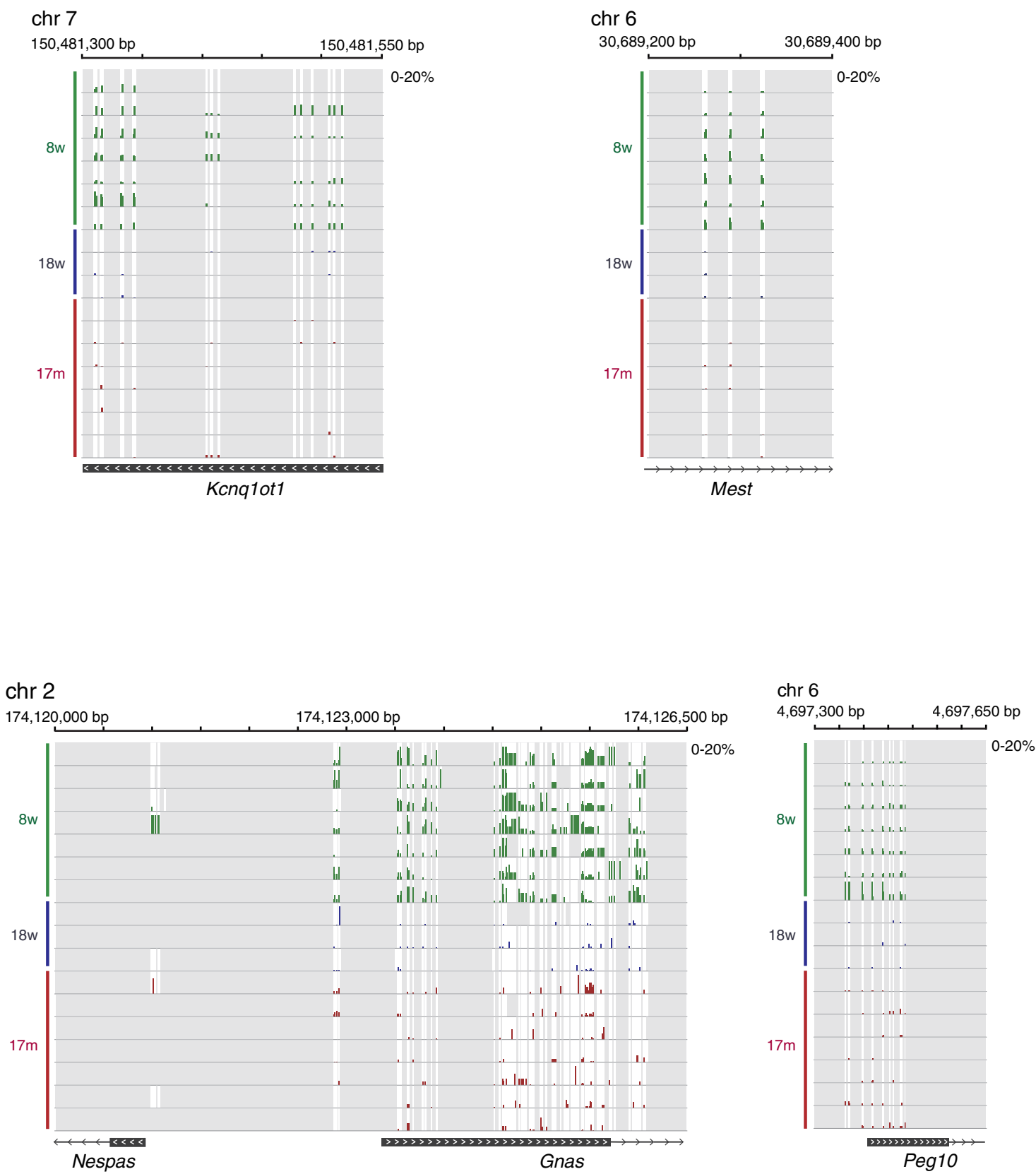

# Supplementary Figure 3\_3 (continued)

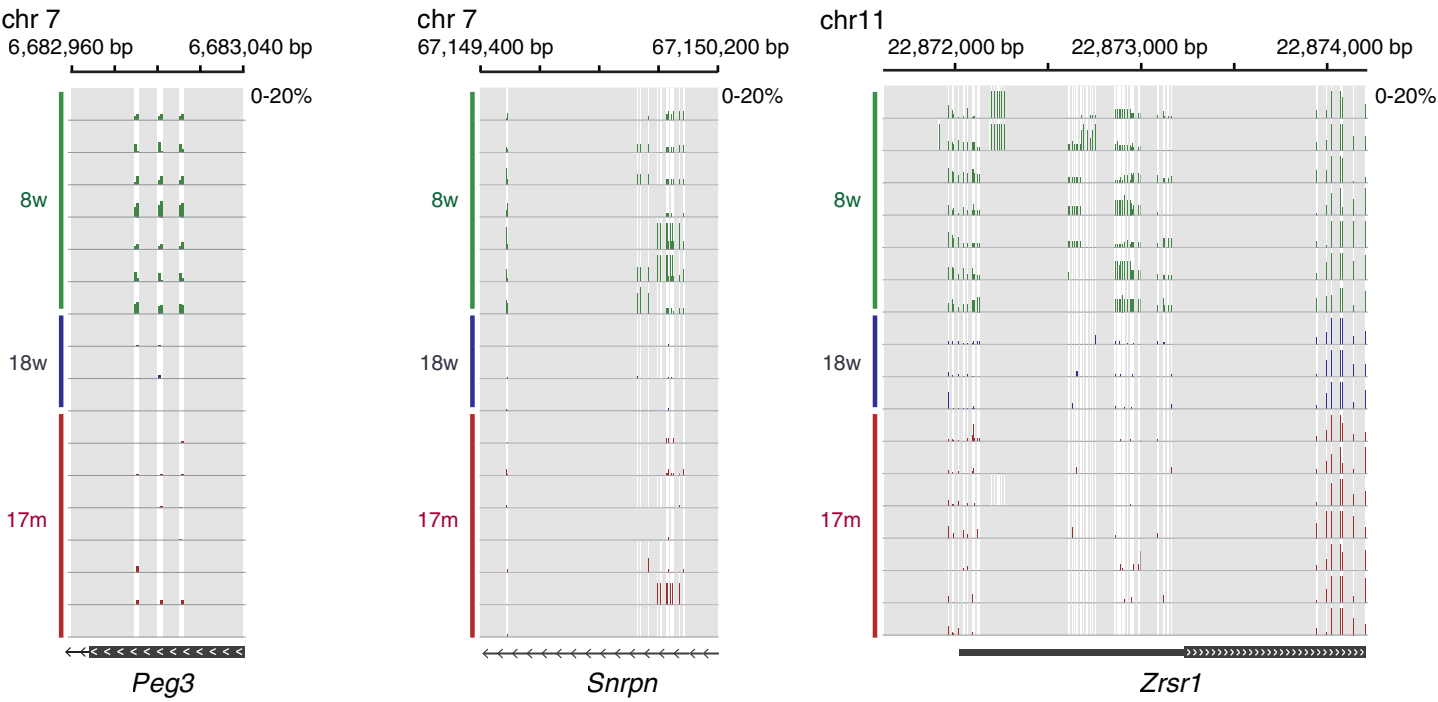

Supplement: S3 Fig — The vertical axis indicates the methylation levels (%). (PDF) [file pone.0167127.s004.pdf]

# Supplementary Figure 4

**A**

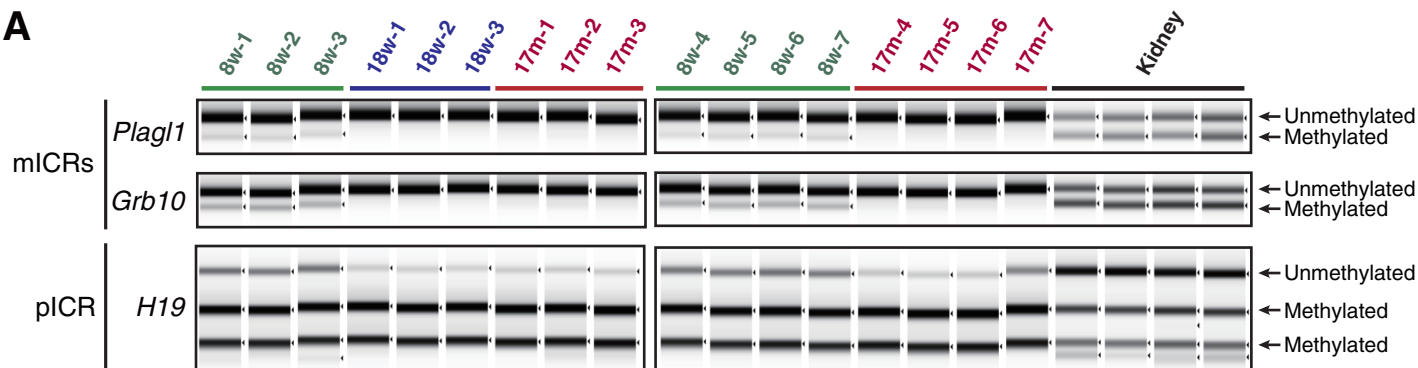

**B**

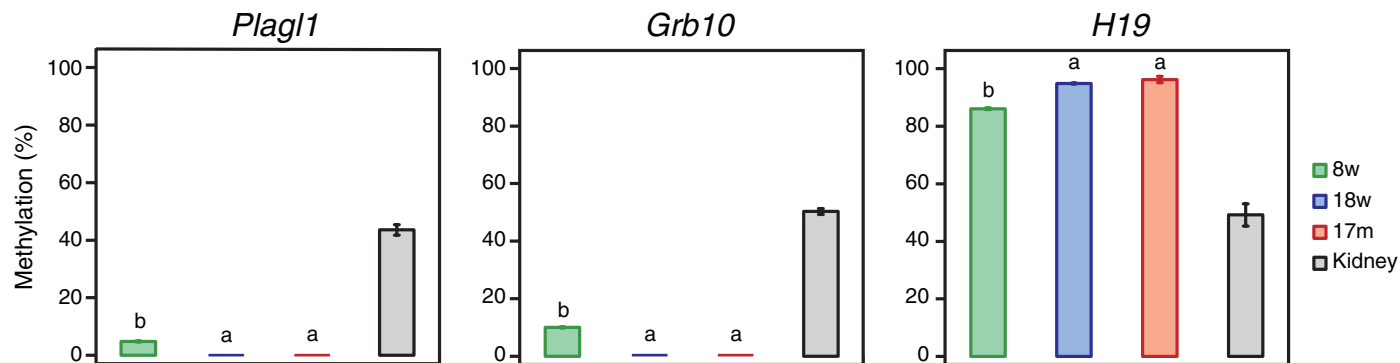

Supplement: S4 Fig — (A) DNA methylation of Plagl1 mICR, Grb10 mICR and H19 pICR in each age sample. Plagl1, Grb10 and H19 amplified by PCR were digested with BstUI or AciI. Kidney DNA was used as a control. (B) Methylation levels of Plagl1 mICR, Grb10 mICR and H19 pICR. Data are shown as mean ± SE. Different letters indicate statistically significant methylation differences (P < 0.05). (PDF) [file pone.0167127.s005.pdf]

# Supplementary Figure 5

**A**

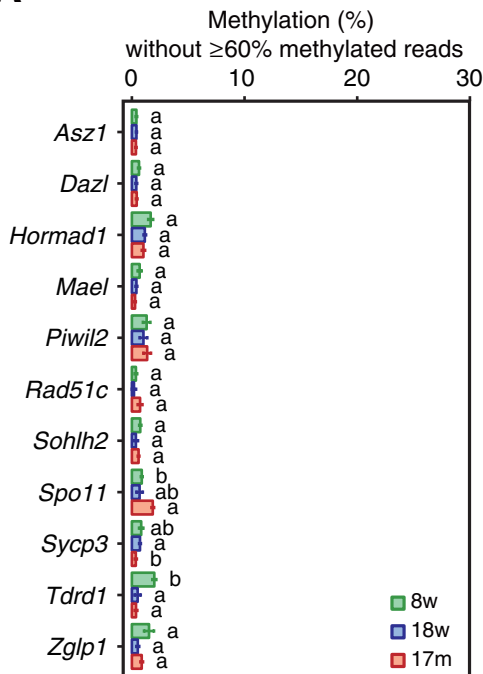

**B**

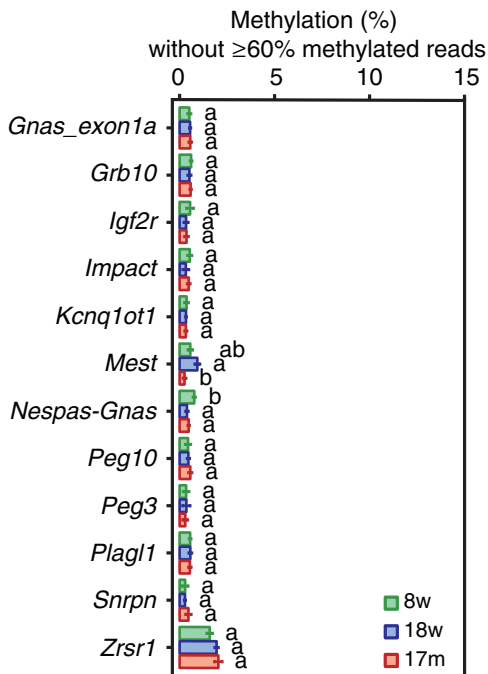

Supplement: S5 Fig — (A and B) Methylation levels of the spermatogenesis-related promoters and mICRs were calculated without ≥60% methylated reads. Data are shown as mean ± SE. Different letters indicate statistically significant methylation differences (P < 0.05). (PDF) [file pone.0167127.s006.pdf]
